# Increasing pelvic incidence is associated with more global sagittal imbalance in ankylosing spondylitis with thoracolumbar kyphosis: an observational retrospective study of 94 cases

**DOI:** 10.1186/s12891-020-03226-0

**Published:** 2020-03-27

**Authors:** Diyu Song, Guoquan Zheng, Tianhao Wang, Dengbin Qi, Yan Wang

**Affiliations:** 1 Medical School of Chinese PLA, No.28 Fuxing Road, Beijing, 100853 China; 2 Department of Orthopedics, General Hospital of Chinese People’s Liberation Army, Beijing, 100853 China

**Keywords:** Ankylosing spondylitis, Pelvic incidence, Sagittal spinopelvic parameters, Global sagittal balance

## Abstract

**Background:**

Ankylosing spondylitis (AS) patients with kyphosis have an abnormal spinopelvic alignment and pelvic morphology. Most studies focus on the relationship of pelvic tilt (PT) or sacral slope (SS) and deformity, and relatively few studies have addressed the relationship between pelvic incidence (PI) and kyphosis in AS patients. The purpose of this study is to analyze the correlation between pelvic incidence (PI) and the spinopelvic parameters describing local deformity or global sagittal balance in AS patients with thoracolumbar kyphosis.

**Methods:**

A total of 94 patients with AS (91 males and 3 females) and 30 controls (27 males and 3 females) were reviewed. The mean age was 36.8 years in AS patients and 34.4 years in controls. Gender ratios and mean age were similar in both group. Sagittal spinopelvic parameters, including PI, PT, SS, thoracic kyphosis (TK), thoracolumbar kyphosis (TLK), lumbar lordosis (LL), sagittal vertical axis (SVA), the first thoracic vertebra pelvic angle (TPA), spinosacral angle (SSA) and spinopelvic angle (SPA) were measured. The same spine surgeons measured all the parameters of the AS and control group. All the sagittal spinopelvic parameters were compared between the groups. The relationship between PI and other spinopelvic parameters was analyzed with Pearson correlation (r) and unary linear regression model.

**Results:**

All the sagittal parameters were found to be significantly different between AS patients and controls. Compared with the control group, the AS patients had significantly higher PI(47.4° vs. 43.2°, *P* < 0.001). Correlation analysis revealed that PI in AS patients was significantly positively correlated with TPA(r = 0.533, R^2^ = 0.284, *P* < 0.001), and negatively correlated with SPA(r = − 0.504, R^2^ = 0.254, *P* < 0.001). However, no correlations were found between PI and SVA, SSA, TK, TLK or LL in AS patients.

**Conclusion:**

This study revealed that increasing PI was significantly correlated with more global sagittal imbalance, not with the local deformity in AS patients with thoracolumbar kyphosis.

## Background

Ankylosing spondylitis (AS) is a chronic, inflammatory rheumatic disease that primarily involves the axial skeleton, which can cause characteristic rigid thoracolumbar kyphosis (TLK) in the latter stage, leading to sagittal spinopelvic imbalance and impairing the patient’s horizontal gaze and activities of daily living [1, 2]. The exact prevalence of kyphosis in AS patients remains unclear. Vosse et al. [3] reported that 45.2% of AS patients had some degree of hyperkyphosis and 81% of them were male. After 30 years of follow-up, Carette et al. [4] found that severe spinal deformity were observed in 9 of the 51 AS patients (18%). For those AS patients with severe kyphotic deformity, corrective spinal osteotomy is usually necessary.

In recent years the sagittal morphology of the spine and pelvis has become one of the most focused topics in spinal deformity research. Pelvic incidence (PI) is an individual and position-independent anatomic spinopelvic parameter [5–8]. It is defined as the angle between the line perpendicular to the sacral plate at its midpoint and the line connecting this point to the axis of the femoral heads [8]. Previous studies have shown that abnormal PI is a risk factor for sagittal imbalance of the spine and may be related to certain spinal pathology [6, 9, 10]. Hanson et al. [9] founded that PI was significantly correlated with the degree of isthmic spondylolisthesis. Cho, et al. [10] demonstrated that higher PI was a risk factor for the development of sagittal imbalance in degenerative lumbar scoliosis after long instrumentation and fusion. PI also determines the ability of the compensation for the spinal deformity by pelvic retroversion [11], which is the major accommodation of kyphotic deformity [12]. For AS patients with kyphosis, the accurate angle required for spinal osteotomy can be calculated based on the value of PI [1]. Besides, PI and lumbar lordosis (LL) mismatching can also be applied in the evaluation of the surgical outcomes in AS patients with kyphosis [13].

Several studies have demonstrated that AS patients with kyphosis have an abnormal spinopelvic alignment and pelvic morphology [14–18]. Most of them focus on the relationship of pelvic tilt (PT) or sacral slope (SS) and deformity, and relatively few studies have addressed the relationship between PI and kyphosis in AS patients. Debarge et al. [14] reported that PI in AS patients was significantly higher compared with normal control group. While Lee et al. [15] found that PI was lower in AS patients and the positive correlation was observed between PI with the spinosacral angle (SSA) [19], a parameter assessing the whole deformity, which indicated that AS patients with lower PI had a greater risk of sagittal imbalance. However, their results also showed that PI was higher in sagittal imbalance group than that in sagittal balance group [15]. Therefore, we consider that a more comprehensive understanding of relationship between PI and AS is needed, which would be useful for assessment of deformity and surgical planning in AS patients with kyphosis. The purpose of this study is to analyze the correlation between PI and the spinopelvic parameters describing local deformity or global sagittal balance in AS patients with thoracolumbar kyphosis.

## Methods

We performed a retrospective study of consecutive patients with AS who underwent spine surgery at our institution between January 2011 and December 2016. All AS patients met the most recent modified New York criteria [20]. The inclusion criteria were (1) Type II (thoracolumbar) kyphosis [21], (2) no scoliosis or with a coronal curve < 10°, and (3) no hip flexion contractures. The exclusion criteria were (1) previous spine, hips or lower extremity surgery, (2) preoperative infection of the spine and (3) preoperative spinal fractures. Therefore, this study included 94 AS patients (91 males and 3 females) with a mean age of 36.8 years old (range 21 to 65). The controls were those asymptomatic adults who underwent full-length spine radiography in orthopedic outpatient clinic just because they suspected they had scoliosis or other spinal deformity. They had no pain in the spine or legs. The physical examination and radiographic results showed that there were no spinal deformity in controls. Thus, there were 30 asymptomatic adults (27 males and 3 females) with a mean age of 34.4 years old (range 22 to 50) in the controls. The present study was approved by the Clinical Research Ethics Committee of our institution.

Full-length spine radiographs including the whole spine and pelvis of AS patients who were standing in a natural position were taken preoperatively. The following radiographical parameters were assessed: PI, the angle between the line perpendicular to the sacral plate at its midpoint and the line connecting this point to the axis of the femoral heads; PT, the angle between the line linking the midpoint of the sacral plate with the femoral head axis and the vertical axis; SS, the angle between the sacral plate and the horizontal line; thoracic kyphosis (TK), the angle between the upper end plate of T4 and the lower end plate of T12; TLK, the angle between the upper end plate of T11 and the lower end plate of L2; LL, the angle between the upper end plate of L1 and the upper end plate of S1; sagittal vertical axis (SVA), the distance between the C7 plumb line (C7PL) and the posterosuperior corner of S1; the first thoracic vertebra pelvic angle (TPA) [22], the angle between the line from the center of the femoral heads to the center of T1 vertebral body and the line from the center of the femoral heads to the center of the superior sacral endplate; SSA [14, 19], the angle between the sacral endplate and the line connecting the midpoint of the sacral endplate and the center of C7 vertebral body; and spinopelvic angle (SPA) [19], the angle between a line from the center of C7 to the center of the sacral endplate and a line from the center of the sacral endplate to the center of the femoral heads. For TK, TLK and LL, the angle was negative if the curve was lordotic and was positive if the curve was kyphotic. Surgimap (New York, NY, USA) was used to measure all the parameters. All measurements were performed twice independently by 3 spine surgeons with an interval of 2 weeks between measurements.

### Statistical analysis

The statistical analyses was performed using IBM SSPS (version 24, IBM Corp.). All continuous variables were presented as the means± standard deviations and were normally distributed. The inter-observer and intra-observer reliabilities of the radiographical measurements were assessed by interclass correlation coefficient (ICC). The spinopelvic parameters and age between the AS patients and the controls were compared with an independent sample t-test. The difference of gender between two groups was studied using a chi-square test. Multivariate ANOVA and Bonferroni post hoc correction for multiple comparisons were used to further analysis the difference of the spinopelvic parameters between two groups. Pearson correlation (r) and unary linear regression model were used to analysis the relationship between PI and other spinopelvic parameters. *P* < 0.05 was considered statistically significant.

## Results

The mean disease duration of AS patients was 15 years (Table 1). Mean age and gender ratios were similar in AS group and control group. The ICC showed excellent inter-observer(0.92–0.95) and intra-observer(0.90–0.94) reliabilities for measurements of spinopelvic parameters. The sagittal spinopelvic parameters of AS patients and controls were presented in Table 1. All the sagittal parameters were found to be significantly different in AS patients and controls before or after correction for multiple comparisons. Compared with the control group, AS patients had significantly higher PI(47.4° vs. 43.2°), PT(38.6° vs. 7.8°), TK(53.9° vs. 32.3°), TLK(33.3° vs. 6.6°), SVA(166.1 mm vs. 0.6 mm), and TPA(47.1° vs. 4.0°), whereas AS patients had significantly lower SS(8.8° vs. 35.4°), LL(− 5.4° vs. -46.3°), SPA(120.5° vs. 173.5°), and SSA(77.8° vs. 126.9°)than those of controls.

**Table 1:** Comparison of the spinopelvic parameters between AS patients and normal controls

	AS group (*n* = 94)	Control group (*n* = 30)	P^a^	P (adjust)^b^
Disease duration (yr)	15.0 ± 7.0			
Sex,M/F	91/3	27/3	0.130	
Age (yr)	36.8 ± 9.6	34.4 ± 8.3	0.225	
PI (°)	47.4 ± 7.5	43.2 ± 4.6	< 0.001*	0.003*
PT (°)	38.6 ± 11.7	7.8 ± 3.2	< 0.001*	< 0.001*
SS (°)	8.8 ± 10.2	35.4 ± 3.7	< 0.001*	< 0.001*
TK (°)	53.9 ± 18.1	32.3 ± 6.2	< 0.001*	< 0.001*
TLK (°)	33.3 ± 12.7	6.6 ± 5.0	< 0.001*	< 0.001*
LL (°)	−5.4 ± 14.2	−46.3 ± 6.4	< 0.001*	< 0.001*
SVA (mm)	166.1 ± 58.1	0.6 ± 15.1	< 0.001*	< 0.001*
SPA(°)	120.5 ± 15.2	173.5 ± 1.1	< 0.001*	< 0.001*
SSA(°)	77.8 ± 13.3	126.9 ± 4.1	< 0.001*	< 0.001*
TPA (°)	47.1 ± 12.5	4.0 ± 1.0	< 0.001*	< 0.001*

*AS* ankylosing spondylitis; *M* male; *F* Female; *PI* Pelvic incidence; *PT* Pelvic tilt; *SS* Sacral slope; *TK* Thoracic kyphosis; *TLK* Thoracolumbar kyphosis; *LL* Lumbar lordosis; *SVA* Sagittal vertical axis; *GK* Global kyphosis; *SPA* spinopelvic angle; *SSA* spinosacral angle; *TPA* the first thoracic vertebra pelvic angle

^a^ Compared with independent sample t-test or chi-square test

^b^ Adjusted by multivariate ANOVA and Bonferroni post hoc correction

*Statistically significant (*P* < 0.05)

The correlations between PI and other spinopelvic sagittal parameters in AS patients and controls were shown in Table 2. For AS patients, PI was found to be significantly positively correlated with TPA(r = 0.533, *P* < 0.001), and negatively correlated with SPA(r = − 0.504, *P* < 0.001). However, no correlations were found between PI and SVA, SSA, TK, TLK or LL in AS patients.

**Table 2:** Correlation coefficient(r) between PI and other sagittal parameters in AS patients and controls

Correlation coefficient(r)	PI			
	AS group	*P* value	Control group	P value
PT	0.506	< 0.001*	0.601	< 0.001*
SS	0.159	0.127	0.722	< 0.001*
TK	0.171	0.100	0.090	0.637
TLK	−0.67	0.522	−0.292	0.117
LL	−0.135	0.194	−0.540	0.002*
SVA	0.090	0.389	−0.163	0.389
SPA	−0.504	< 0.001*	−0.296	0.112
SSA	−0.11	0.919	0.803	< 0.001*
TPA	0.533	< 0.001*	0.265	0.157

*AS* ankylosing spondylitis; *PI* Pelvic incidence; *PT* Pelvic tilt; *SS* Sacral slope; *TK* Thoracic kyphosis; *TLK* Thoracolumbar kyphosis; *LL* Lumbar lordosis; *SVA* Sagittal vertical axis; *GK* Global kyphosis; *SPA* spinopelvic angle; *SSA* spinosacral angle; *TPA* the first thoracic vertebra pelvic angle

*Statistically significant (*P* < 0.05)

**Fig. 1:**
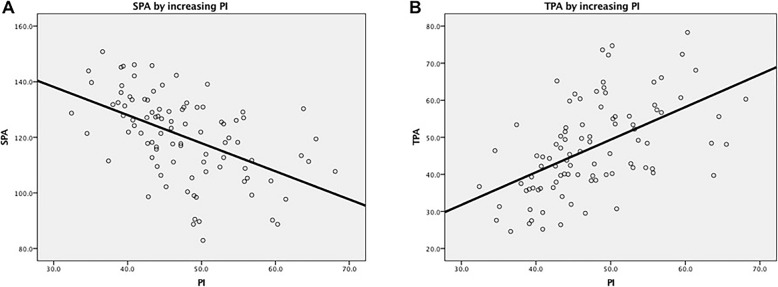
**a**, Scatter diagram of SPA and PI. **b**, Scatter diagram of TPA and PI. SPA indicates spinopelvic angle; PI, Pelvic incidence; TPA, T1 pelvic angle

Unary linear regression analysis was also performed to showed that a tendency for positive direct linear association between PI and TPA(R^2^ = 0.284, *P* < 0.001), and a tendency for negative direct linear association between PI and SPA(R^2^ = 0.254, *P* < 0.001) in AS patients (Table 3, Fig.1).

**Table 3:** Linear regression model for PI with TPA and SPA in AS patients

	Unstandardized coefficients	Standardized coefficients	R^2^ of model	*P*
TPA	0.323	0.533	0.284	< 0.001*
SPA	−0.251	−0.504	0.254	< 0.001*

*AS* ankylosing spondylitis; *PI* Pelvic incidence; *SPA* spinopelvic angle; *TPA* the first thoracic vertebra pelvic angle

*Statistically significant (*P* < 0.05)

## Discussion

In the present study, spinal malalignment in AS patients was observed. AS patients presented higher PT, TK, TLK, SVA, and TPA, and lower SS, LL, SPA and SSA, which indicated retroversion of the pelvis, more local deformity and greater global imbalance. These results were consistent with previous published studies [14, 15, 17]. We also noticed that in our study the mean value of PI(47.4°) in AS patients with kyphosis was statistically higher than that of controls(43.2°). Debarge et al. [14] found the mean value of PI was 61.9 ° in AS group and 50.6 ° in the control group by analyzing 28 AS patients with fixed kyphosis and 154 asymptomatic volunteers. However, Lee et al. [15, 17] found that AS patients had a lower PI compared to normal controls. The difference might be mainly due to different AS and control population included. A previous study confirmed that Chinese population had lower PI compared with Caucasian individuals [23]. Besides, extremely wide variations have been showed in the value of PI, which ranged from 35° to 85° [24]. Therefore, whether the value of PI was higher or lower in AS still needs more investigations to be confirmed furtherly.

To date, correlation between sagittal spinopelvic parameters and the development of spinal disorders in patients with AS is not clearly confirmed yet. Debarge et al. [14] proposed that the patients with a high PI would have more kyphotic deformity than those with a low PI if they had the same C7 plumb line position. However, Lee et al. [15] confirmed that AS patients with a lower PI had a greater risk of sagittal imbalance. In their both investigations, only SVA and SSA were analyzed as parameters assessing the whole deformity. So, we wondered what was the real potential role of PI in the pathology of AS with kyphosis. To clarify this question, we evaluated the relationship between PI and the spinopelvic parameters describing local deformity or global sagittal balance in AS patients with thoracolumbar kyphosis.

**Fig. 2:**
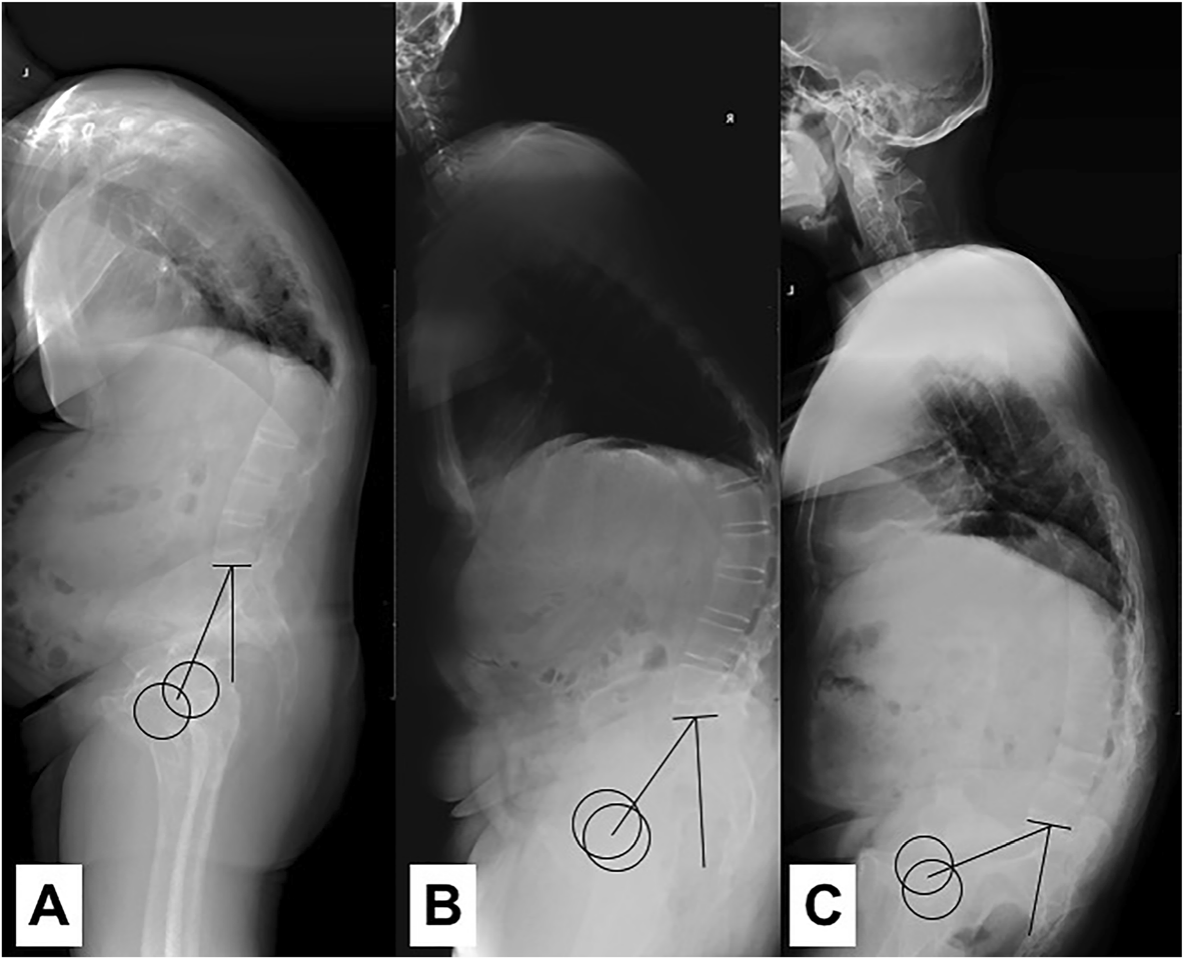
Lateral X-rays of AS patients showed increasing PI was correlated with more global sagittal imbalance (larger TPA, lower SPA). In Fig.2-**a** the PI, TPA and SPA were 32.4°, 36.7° and 128.7° respectively. In Fig.2-**b** the PI, TPA and SPA were 45.9°, 60.4°and 123.3° respectively. In Fig.2-**c** the PI, TPA and SPA were 56.8°, 66.1° and 99.2° respectively

In our study, the parameters indicating global sagittal balance included TPA, SPA, SVA and SSA. The results showed that in AS patients with thoracolumbar kyphosis increasing value of PI was significantly correlated with more global sagittal imbalance (larger TPA, lower SPA) and there was no association between PI and the local deformity (Fig. 2). However, we also found that PI in AS was not significantly correlated with SVA or SSA, which are also the spinopelvic parameters usually used to measure the whole sagittal deformity. There might be several reasons for this. First, the value of SVA is greatly influenced by pelvic compensation or patients’ postures [22, 25]. For this reason, Van Royen et al. [25] suggested that it was not suitable to use SVA to measure the sagittal imbalance in AS from a standing full-length spine radiograph. Second, SSA strongly correlates with SS in a healthy population [19]. Normal individual with higher PI may has higher SSA, which was confirmed by the dates of control group in our study. Third, TPA or SPA is independent of position and pelvic retroversion. TPA also combines the information from both SVA and PT [22]. Besides, SSA or SPA decreases as the deformity increases, while TPA increases as the deformity increases [19, 22]. Therefore, it may be better to use TPA or SPA to evaluate the global sagittal alignment of spinal deformity. However, the reason why increasing PI was associated with more global sagittal malalignment was not confirmed in this study. We believe that there would be other risk factors of global sagittal malalignment for AS patients with kyphosis, which need more investigations.

It had been confirmed that PT、SS and LL were correlated with PI in normal population [26, 27]. However, there was no such relationship between LL or SS and PI in our AS patients. We believe that it is the spinal kyphotic deformity that changes the relationship between PI and other parameters. The exact reason and mechanism were still unconfirmed in our study. The spinal deformity of AS patients is more complex and there are still some unknown things about the pathogenesis and development of deformity. On the other hand, TPA was not correlated with PI in controls in our study. However, Zhou et al. [27] found that TPA was significantly associated with PI in 218 asymptomatic adults. The reason might be the difference of population included in both studies and the number of our control group was smaller. The association between increasing PI and larger TPA in AS patients with thoracolumbar kyphosis might be significant for the surgical design. Protopsaltis et al. [28] also found that TPA was significantly correlated with PI in adult spinal deformity and suggested that the targets for sagittal spinal alignment increase with increasing PI.

There are some limitations in our study that require consideration. First, the present study is essentially a retrospective review. Therefore, a prospective study with a long follow-up may be needed to further confirm the role of PI in the development of deformity of AS. Second, AS patients were examined by X-ray in a natural standing position, however some patients with severe kyphosis might stood with the knees in natural flexion in order to compensate for sagittal imbalance. Thus, some parameters depending on position, such as SVA, were not accurate. Furthermore, the number of normal controls was relatively small and only AS patients with severe kyphosis were included. As such, selection bias may exist in our study. The result in our study should not be generalized to the whole AS population.

## Conclusions

This study showed that the spinopelvic sagittal parameters were significantly different in AS patients and controls. Furthermore, the value of PI in AS patients with kyphosis was significantly higher than that of controls. Correlation analysis revealed that increasing PI was significantly correlated with more global sagittal imbalance, not with the local deformity in AS patients with thoracolumbar kyphosis.

## Data Availability

The patients’ data were collected in the Chinese PLA General Hospital. The datasets used and/or analyzed during the current study are available from the corresponding author on reasonable request.

## Abbreviations

AS: Ankylosing spondylitis
PI: Pelvic incidence
PT: Pelvic tilt
SS: Sacral slope
TK: Thoracic kyphosis
TLK: Thoracolumbar kyphosis
LL: Lumbar lordosis
SVA: Sagittal vertical axis
C7PL: The C7 plumb line
TPA: The first thoracic vertebra pelvic angle
SSA: Spinosacral angle
SPA: Spinopelvic angle
ICC: Interclass correlation coefficient
